# Prevalence and associated factors of scabies in Ethiopia: systematic review and Meta-analysis

**DOI:** 10.1186/s12879-020-05106-3

**Published:** 2020-05-27

**Authors:** Abebaw Gedef Azene, Abiba Mihret Aragaw, Gizachew Tadesse Wassie

**Affiliations:** 1 Department of Epidemiology and Biostatistics, School of Public health, College of Medicine and Health Science, Bahir Dar University, Bahir Dar, Ethiopia; 2 Department of Statistics, College of Natural and Computational Science, Debre Markos University, Debre Markos, Ethiopia

**Keywords:** Prevalence, Associated factors, Scabies, Meta-analysis, Systematic review, Ethiopia

## Abstract

**Background:**

Scabies is an infectious disease that affects the skin caused by the *mite Sarcoptes scabiei* and it transmitted through close personal contact. Even though it is easily treatable disease, its prevalence is high and continuous as neglected tropical disease of resource-poor settings, and particularly affects young age groups. Despite of these facts, studies conducted in Ethiopia regarding to the prevalence and associated factors for scabies infestation have been highly variable and didn’t well compiled. Due to that, the aim of this systematic review and meta-analysis was to estimates the overall prevalence of scabies and associated factors in all age groups in Ethiopia.

**Methods:**

International databases (PubMed/PMC/Midline, EMBASE, CINAHL, Web of Science, Google Scholar, Google and Science Direct) were systematically searched from December 1, 2019, to January 18, 2020. All observational studies noted the prevalence of human scabies and associated factors in Ethiopia were included. Two authors (AG and G.T) independently extracted all necessary data using a standardized data extraction format. The data which is extracted each study were analyzed using STATA Version 14.1. Heterogeneity among the included studies was assessed through the Cochrane Q test statistics and I^2^ test. Lastly, a random effects meta-analysis model was computed to fix overall prevalence and associated factors of scabies.

**Results:**

Twelve studies were included in this meta-analysis after 410 articles retrieved. Of these, eight studies were analyzed for prevalence estimation. The overall prevalence of scabies infestation was 14.5% (95%CI: 1.5, 27.6%) in Ethiopia. Furthermore, the subgroup analysis revealed the highest prevalence was 19.6% in Amhara region. A person from a large family size (OR: 3.1, 95% CI: 1.76, 5.67), and sharing a bed (OR: 3.59, 95%CI: 2.88, 4.47) were significantly associated with scabies.

**Conclusion:**

This study revealed the prevalence of scabies infestation was 14.5% in Ethiopia which was high. Persons from high family size and any contact with scabies case were factors associated with scabies.

## Background

Scabies is an infectious disease that affects the skin caused by the *mite Sarcoptes scabiei* and it transmitted through close personal contact [1, 2]. The manifestation begins with itching, which results in complications of bacterial infections [1, 3]. These complications may include local skin infection, abscesses, kidney and heart disease [2].

Scabies and its burdens are often regarded as a problems of people living in low and middle income countries, and commonly affects young and elder age persons [1, 4]. However, scabies have several effective treatment options [5], prevention and control in the population are challenging because of the high levels of re-infestation that can occur through community and personal contacts [4, 5]. The spread of scabies are high in the overcrowding area [3].

The prevalence of scabies is ranging from 0.3 to 46% and the point estimate is around 147 million worldwide [5, 6] . There is a high prevalence in low and low-middle income countries [4, 7]. Its prevalence in Sub-Saharan Africa was varied up to 33.7% [8–10]. The prevalence of scabies in Nigeria is about 4.7% up to 65% [11, 12].

Scabies is one of public health concerns among communicable disease in Ethiopia, especially disadvantaged people like streets, migrants and poorer [5, 8]. The magnitude of scabies infestation in Ethiopia was varied which ranged from 2.5 to 78% and inconsistent [13, 14]. To our best knowledge, there is no study which estimates a pooled prevalence of scabies in Ethiopia. The aim of this study was to estimate the pooled prevalence and associated factors of scabies in all age groups. This systematic review and meta-analysis may use an alarm for policy makers to improve prevention and control strategies of the disease.

## Methods

### Search strategy and identification of studies

We have searched studies which reported the prevalence and factors associated with scabies in Ethiopia by following PRISMA guidelines. To identify potentially relevant studies, all-embracing search was performed from the following electronic database i.e. PubMed/PMC/Medline, EMBASE, CINAHL, Web of Science, Google Scholar, Google, Science Direct and Cochran library. Additionally, unpublished papers were searched like Addis Ababa University digital library. The keywords used to search studies were “prevalence”, “associated factors”, “determinant factors”, “scabies” and “Ethiopia” using OR or AND conjunction with English language restriction. Gray literatures were searched from a reference list of studies which included in this systematic review and meta-analysis. Selected articles were retrieved and managed using Endnote X9. The search was conducted from December 1, 2019 up to January 18, 2020. All studies which published between January 01, 2000 and January 18, 2020 were included.

### Inclusion and exclusion criteria

#### Inclusion criteria

All published and unpublished observational studies conducted in Ethiopia, which reported prevalence or associated factors of scabies with English language were considered. Studies, which published between January 01, 2000 and January 18, 2020 were included.

#### Exclusion criteria

Full text articles were searched for only eligible studies. Articles unable to access their full text were excluded. For the first outcome case control study designs without defining population was excluded.

### Outcome variable

A primary outcome of this systematic review and meta-analysis was a prevalence of human scabies in all age groups. The prevalence of scabies were calculated the proportion of people who infected by scabies among all participants in the study.

A second outcome of this study was identifying the major factors associated with scabies. Identified factors associated with scabies for each study with a log odds ratio were extracted or calculated from the selected studies. Factors associated with scabies included in this study were family size, frequency of bath, any contact (sharing bed, cloth and skin contact) with scabies case and hand washing with soap.

### Data extraction

In this review, two authors (AG and GT) identified articles and extracted relevant data independently. Data were extracted from full text studies. The extracted data were primary author name, publication year, regions where the study was conducted, study area, sample size, study design, prevalence with 95% confidence intervals, the response rate and odds ratio or 2 × 2 contingency table for the selected each factor was extracted on the reports of original studies. Any disagreement between the two authors due to inclusion and data collection was solved by discussion and consulting with the third author (A.M).

### Quality assessment

Two authors (AG and GT) independently assessed the quality of each original study using the quality assessment tool. All included published and unpublished studies were assessed for inclusion using their title and abstract. To assess the quality of studies, the Joanna Briggs Institute meta-analysis of Statistics Assessment and Review Instrument (JBI-MAStARI) quality assessment checklist for prevalence, cross sectional and case control study was used for each study [15]. A studies which has five and above quality score were included for both study design. A study, which has five and above quality score were considered at low risk of bias, and at high risk of bias which scored less than five. During the quality assessment of the studies, the agreement of between the two authors (AG and GT) was tested using Kappa (0.86).

### Data analysis and assessment of risk bias

Microsoft Excel format was used to manage the extracted data and analyzed using STATA version 14.1 Software. The extracted data were presented using forest plot which shows point estimates of study effects and their confidence interval [16]. The size of the box indicated the sample size or weights and the horizontal line shows confidence interval or the precision of the study [17]. It also gives highlight information about heterogeneity.

We assessed the heterogeneity of the included studies using the Cochran Q and I^2^ test [18]. The value of I^2^ greater than 50 was considered as the existence of heterogeneity in the studies [19, 20]. A random effect meta-analysis was used due to the presence of heterogeneity [21, 22]. To identify the source of this heterogeneity and the distribution, subgroup and sensitive analysis was conducted [23]. Funnel plot and Eggers test were used to check the presence and significance of publication bias [23, 24].

## Results

### Description of identified studies

**Fig. 1:**
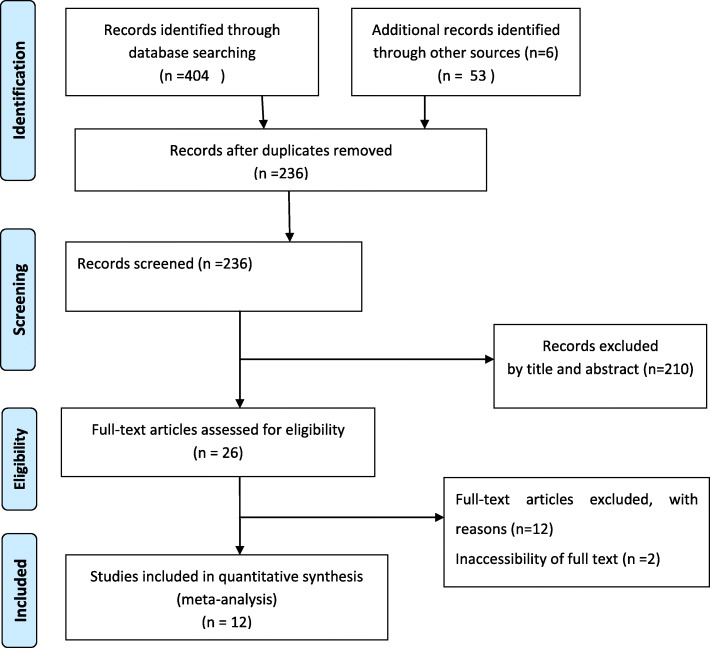
Follow diagram of study selection for systematic and Meta-analysis of scabies in Ethiopia

In this systematic and meta-analysis study, a total of 410 titles and abstracts was searched using previously noted electronics databases. Of these potentially relevant articles, 164 studies were excluded because of duplication. Two hundred ten studies were excluded after detail reviewed of their title and abstract as they did not report either prevalence or associated factors of human scabies infestation in Ethiopia. Furthermore, two articles due to inaccessibility of full text [13, 25], Twelve articles were not conducted on human or did not satisfy the minimum criteria were excluded. Finally, twelve studies were included in this systematic review and meta-analysis. Of those included studies, eight studies were estimated the prevalence [8, 14, 26–31] and eight studies were identified associated factor of scabies [14, 27, 28, 31–35] Fig. 1.

### Characteristics of the included studies

In this review, seven studies which were institution and community based unmatched case control study design, whereas five of them were cross-sectional. All of those included studies were done after 2016. From 8 studies, 1,178,489 study participants were included to estimate the pooled prevalence. The range of the sample size of these studies was 96 to 1,125,770. Eight studies were reported the associated factors of human scabies in Ethiopia. Regarding to the prevalence of scabies, the smallest was 2.5%, which was reported a study conducted in SNNPR and the highest was 35%, which reported a study conducted in Amhara regional state [14, 31]. In this review, the studies were found from only two Ethiopian regions out of nine regions and two administrative cities. Eight studies were from Amhara region and the remaining four studies were from SNNPR Table 1.

**Table 1:** Descriptive summery of 12 studies included in meta-analysis of scabies in Ethiopia

Author	Publication year	Region	Study area	Sample size	Study design	Prevalence (%)	Response rate (%)
Dagne et al. [27]	2019	Amhara	Dabat	494	Institutional based cross-sectional	9.3	91.84
Sara et al. [28]	2018	SNNPR	Badewachow	41,287	cummonity unmached case-control	11	–
Wochebo, et al. [14]	2019	SNNPR	Kechabirra	9720	Unmached case-control	2.5	100
Ejigu, et al. [32]	2019	SNNPR	Damboya	711	Unmached case-control	–	
Enbiale, et al. [8]	2018	Amhara	Amhara	112,570	Census	33.7	–
Melat [33]	2019	Amhara	Habru	300	institution based unmached case control	–	100
Yassin, et al. [34]	2017	Amhara	Gondar Town	96	Unmached case-control	–	100
Tegegne, et al. [29]	2018	Amhara	Borumeda	385	Institutional based cross sectional	15.9	100
Aynalem, et al. [26]	2017	Amhara	Fnoteselam Hospital	317	Institutional based cross sectional	4.1	95.3
Nurie [35]	2017	Amhara	Dembiya	120	Unmached case-control	–	–
Alebachew, et al. [31]	2020	Amhara	Addet Tawn	173	Unmached case-control	35	–
Walker, et al. [30]	2017	SNNPR	Ademe	343	cross sectional	5.5	100

### Prevalence of scabies infestation

**Fig. 2:**
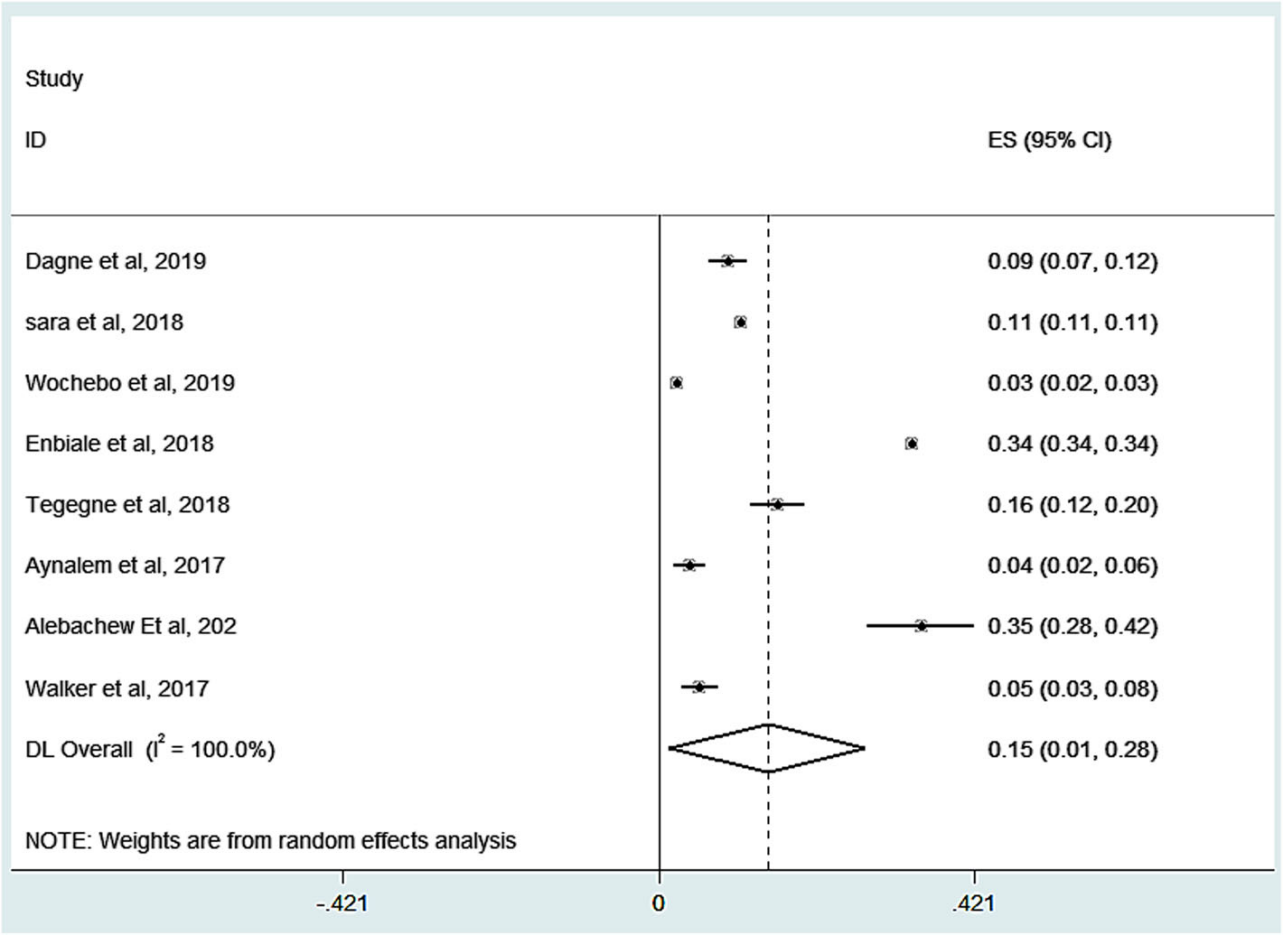
Forest plot for the prevalence of scabies in Ethiopia

The prevalence of scabies in this review were ranging from 4.1 to 35% [26, 31]. We observed that there was a large variation of the prevalence among different studies. Due to high heterogeneity across the included studies in the fixed effect model, random effect meta-analysis model was performed (I^2^ = 99.99%, with Q Cochran *p*-value < 0.001). As a result of random effect model, thus eight studies revealed that a pooled prevalence of scabies among all age groups in Ethiopia was 14.5% (95%CI: 1.5, 27.6%) Fig. 2.

### Subgroup analysis

In this systematic review and meta-analysis study, subgroup analysis was conducted using region where the study conducted, sample size and age group. As a result; the highest pooled prevalence was obtained in Amhara region, which was 19.5% (95% CI: 3.6, 35.4). The highest pooled prevalence of scabies was obtained in younger age groups than the elder age which, was 19.5% (95% CI: 0, 41.9) Table 2.

**Table 2:** Subgroup analysis of the pooled prevalence of scabies in Ethiopia

Variables	Subgroup	No Studies	Event	Total	Prevalence (%)	I^2^(%)	P-value
Region	Amhara	5	379,191	1,127,139	19.6(3.6, 35.4)	99.65	< 0.001
	SNNP	3	4815	51,350	6.3(0.0, 13.1	99.87	< 0.001
Sample size	< 440	4	164	1218	14.4(5.8,23.0)	99.16	< 0.001
	> 440	4	383,842	1,177,271	14.1(0.0,32.6)	99.99	< 0.001
Age in year	< 15	4	281,138	600,153	19.5(0.0, 41.9)	99.98	< 0.001
	> 15	2	102,442	567,521	11.4(0.0,25.6)	99.99	< 0.001

### Sensitivity analysis

We conducted a sensitivity analysis using leave-one-out method to identify possible source of heterogeneity in the estimating of the pooled prevalence of scabies infestation in Ethiopia. As a result, we found that the pooled prevalence didn’t depend on the outcome of a single study and robust. After removal of a single study stepwise, the pooled prevalence of scabies ranged from 11.0% (95% CI; 6.4, 15.5) to 16.3% (95% CI; 4.2, 28.3) Table 3.

**Table 3:** Sensitivity analysis for the prevalence of scabies infestation in Ethiopia

Excluded studies	Prevalence (%)	95% CI	I^2^ (%)	p-value
Dagne, et al., 2019 [27]	15.3	1.3, 29.3	99.9	< 0.001
Sara, et al., 2018 [28]	15.1	0.0, 31.3	99.9	< 0.001
Wochebo, et al.,2019 [14]	16.3	4.2, 28.3	99.9	< 0.001
Enbiale, et al., 2018 [8]	11.0	6.4, 15.5	99.6	< 0.001
Tegegne, et al., 2018 [29]	14.4	0.3, 28.4	99.9	< 0.001
Aynalem, et al., 2017 [26]	16.0	2.1, 30.0	99.9	< 0.001
Alebachew, et al., 2020 [31]	11.7	0.0, 25.7	99.9	< 0.001
Walker, et al., 2017 [30]	15.8	1.8, 29.9	99.9	< 0.001

Furthermore; we have conducted a sensitivity analysis by the JBI quality score, which is categorized as “good quality” (JBI quality score equal to or above 5) and “low quality” (JBI quality score less than 5). We found that only one study, which has low quality in this review (JBI quality score = 4) [14]. Consequently, the pooled prevalence of scabies was 16.3% (95% CI; 4.2, 28.3) after removing a study which has low quality. This result is almost the same with an overall prevalence of scabies (15% (95% CI: 1, 28%)).

### Factors associated with scabies

In this systematic review and meta-analysis, family size, frequency of bath, any contact with scabies person and hand washing with soap were tested for associations with scabies. A separated meta-analysis was conducted for each factor.

### Association of family size with scabies infestation

**Fig. 3:**
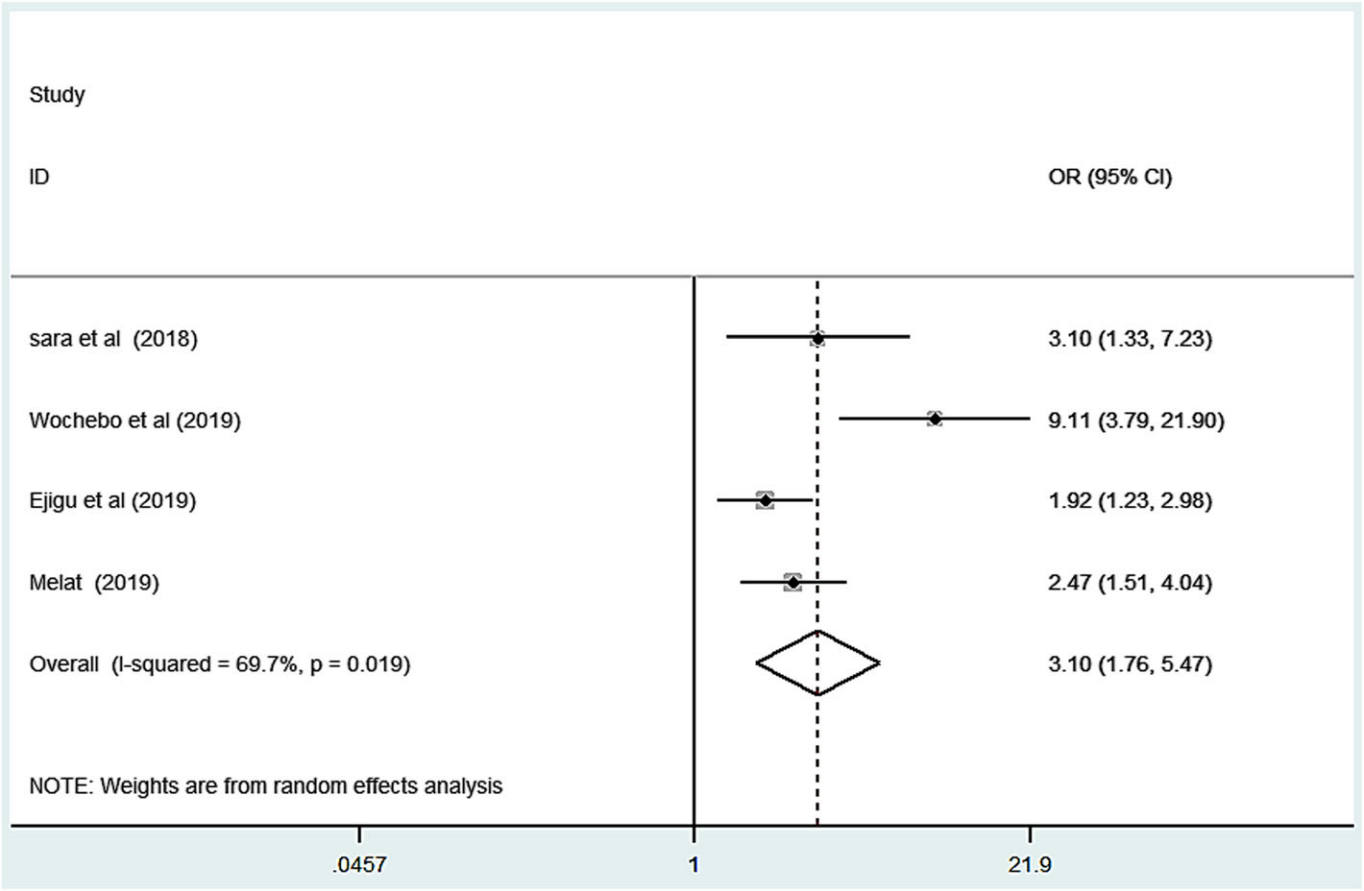
Forest plot for odds ratio of the association of family size with scabies in Ethiopia

**Fig. 4:**
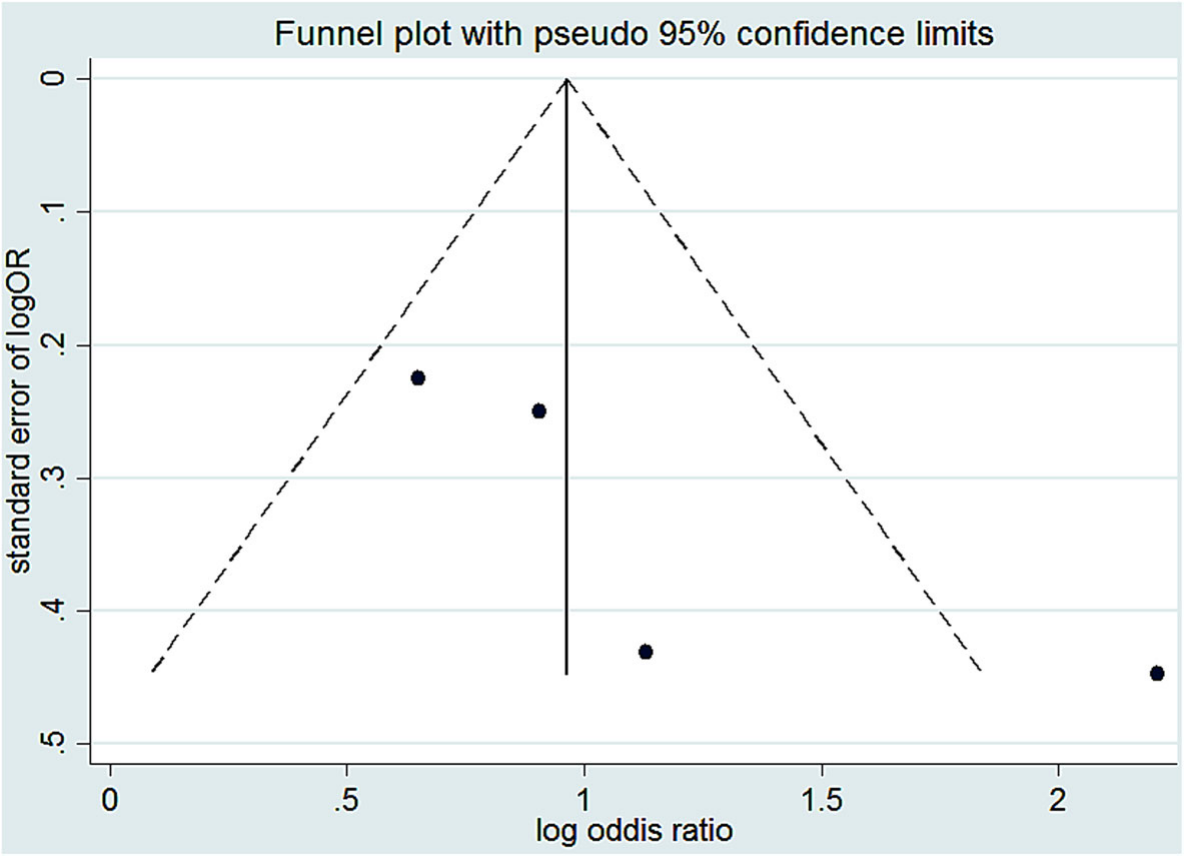
Funnel plot logOR with standard error of logOR of family size with scabies in Ethiopia

A total of four studies were included to estimate the association between family size and scabies [14, 28, 32, 33]. As we see from Fig. 3, high heterogeneity (I^2^ = 69.7% and *p* = 0.019) was observed; due to that meta-analysis with a random effect model was computed. Then wards, there was a statistically significant association between family size and scabies infestation. The pooled odds ratio showed that the odds of scabies from a family having more than five members were 3.1 times higher compared to a family having less than five member counterparts (OR: 3.1, 95% CI: 1.76, 5.67). A funnel plot of publication bias was somehow seems symmetric Fig. 4. Publication bias also assessed using Egger’s tests and the test indicated that there was a low possibility of publication bias with *p*-value of 0.15.

### Association between frequency of bath and scabies infestation

**Fig. 5:**
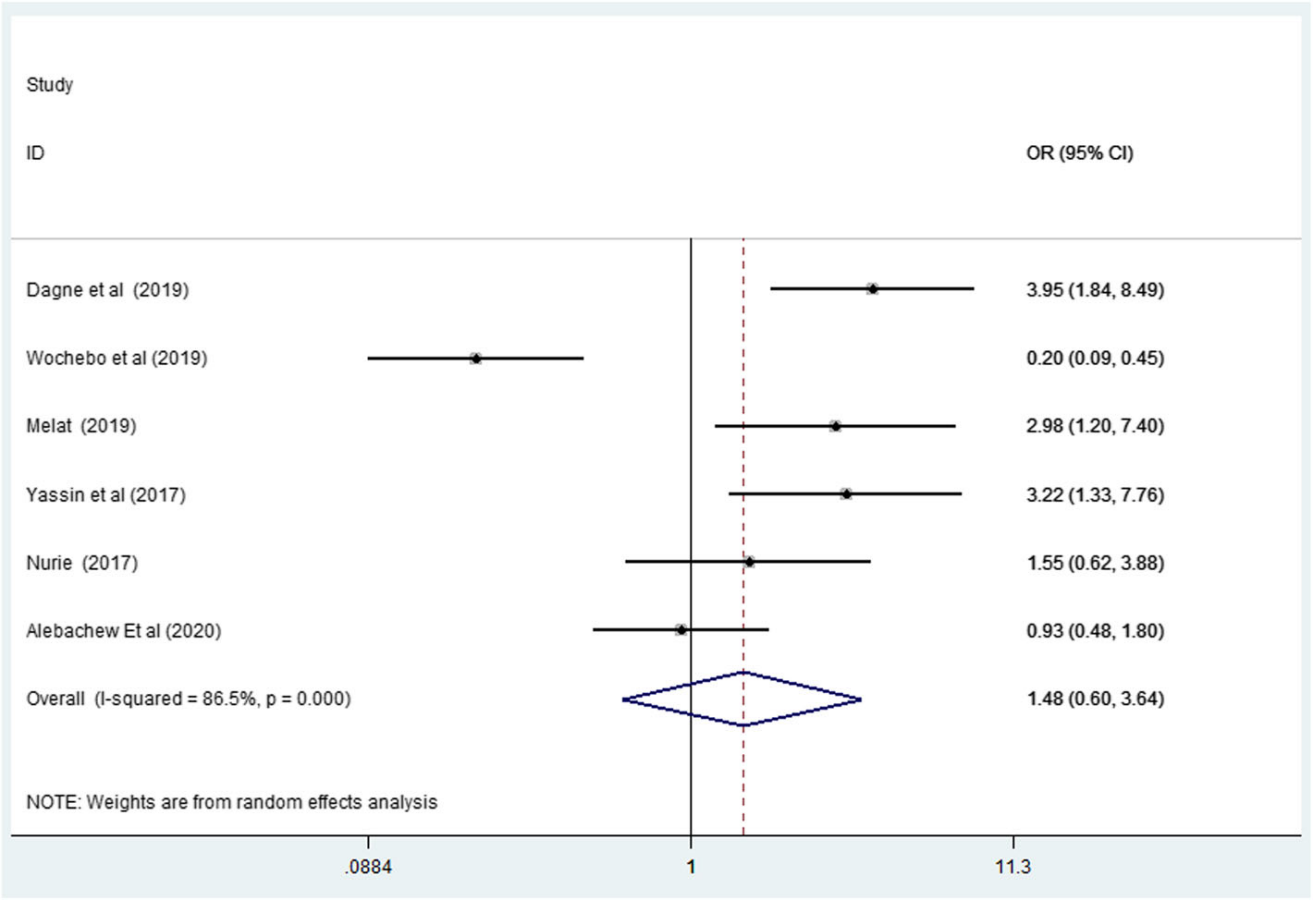
Forest plot for odds ratio of frequency of bath per week with scabies in Ethiopia

To determine the association between frequency of bath and scabies infestation, six studies were included. The result indicated that there was high heterogeneity between studies (I^2^ = 86.5%; *p* < 0.001). The random effect model of a pooled odds ratio of frequency of the bath were not statically significant (OR: 1.47, 95% CI: 0.59, 3.64) Fig. 5.

### Association between any contact with scabies case and scabies infestation

**Fig. 6:**
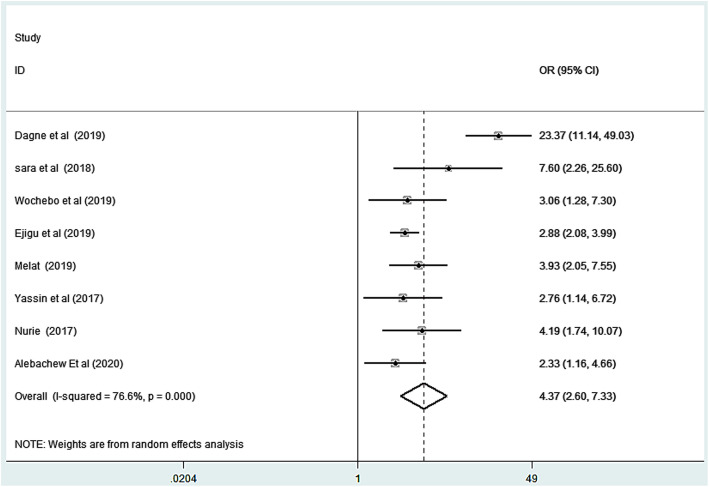
Forest plot for odds ratio of sharing cloth or bed with scabies person and scabies in Ethiopia

**Fig. 7:**
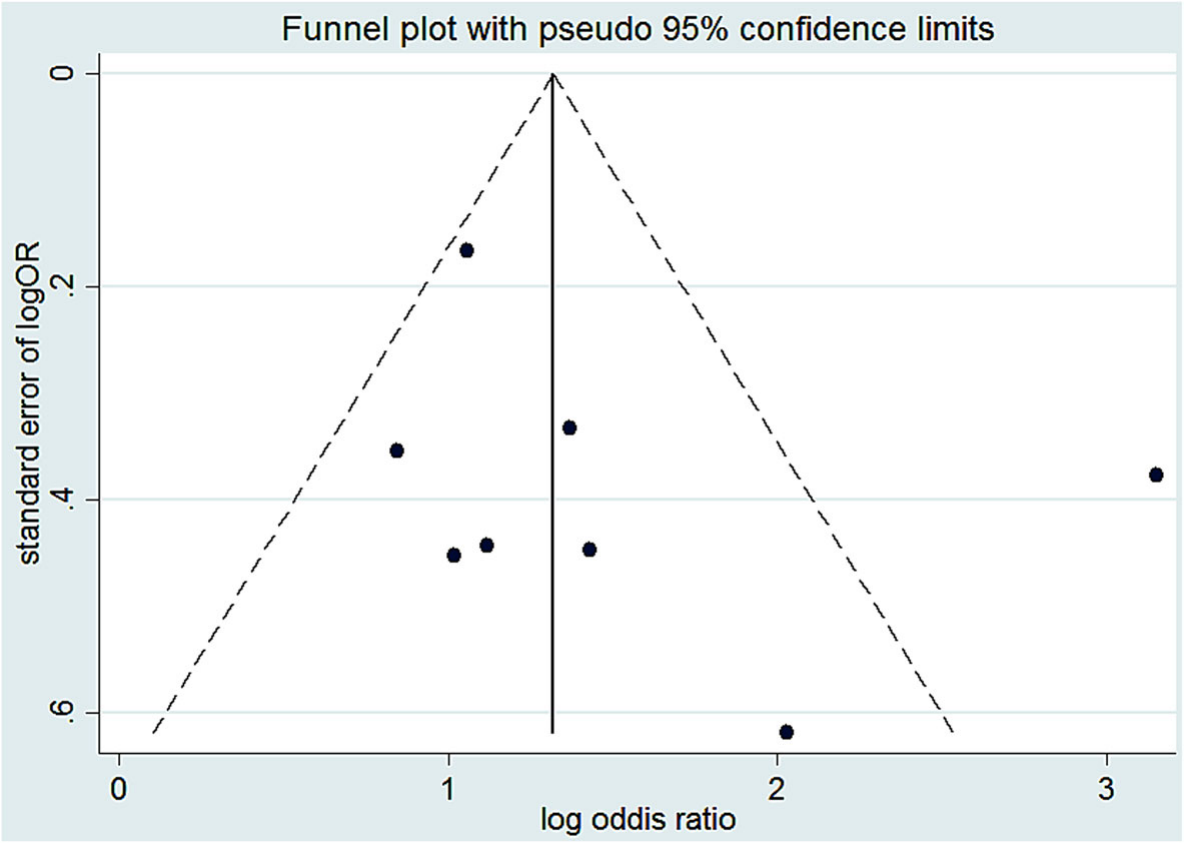
Funnel plot of sharing/sleeping with scabies and scabies in Ethiopia

To determine the association between any contact with scabies person and scabies infestation, eight studies were included. The result of this meta-analysis indicated that there was heterogeneity (I^2^ = 76.6%; *p* < 0.001). Due to this reason, a random effect meta-analysis model was performed. The pooled odds ratio of a random effect model indicated that those who had contact with scabies person were 3.58 times more likely to be infested with scabies as compared to those had no contact (OR: 3.59, 95% CI: 2.88, 4.47). Funnel plot for publication bias was somehow symmetric. In addition to this, the Egger’s tests statistics of publication biases showed that there was no statistically significant publication bias (*p*-value =0.35) Figs. 6 & 7.

### Association of washing hand with soap and scabies infestation

**Fig. 8:**
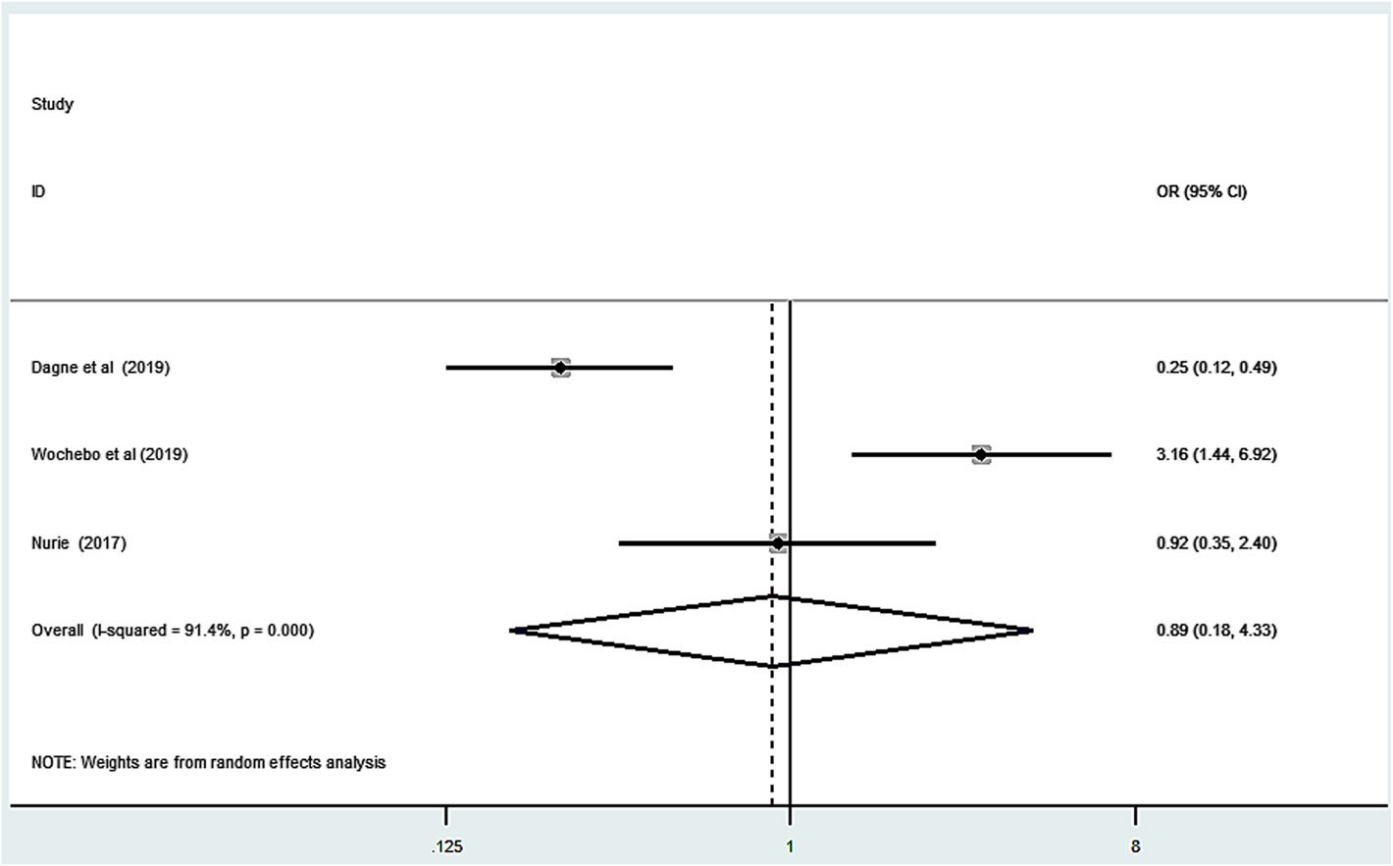
Forest plot the association between hand washing without soap and scabies in Ethiopia

Finally, we have assessed the association of washing hands with soap and scabies infestation using random effect model because of heterogeneity. Three studies were included in the model [14, 28, 35]. The overall pooled odds ratio result of this study revealed that washing hands with soap had no statistically significant association with scabies infestation (OR: 0.89, 95%CI: 0.57, 1.40) Fig. 8.

## Discussion

Scabies is one of the neglected major public health problems in developing countries including Ethiopia. Estimating the pooled prevalence of scabies and its associated factors in the country may contribute to informing policy makers to take remedial action. As far as our knowledge this is the first systematic review and meta-analysis study to determine the overall prevalence of scabies infestation and its associated factors in the country.

This meta-analysis revealed that 14.5% (95%CI: 1.5, 27.6%) of persons was a scabies infestation in Ethiopia. The subgroup analysis showed that Amhara region had a highest prevalence of scabies and the younger age group was more infected. This result was lower than the prevalence study conducted in Solomon Island (19.2%) and Fiji (23.6, 36.4%) [36, 37]. The possible reason might be due to family size or fertility rate variation which is lower in Ethiopia than those countries [38–40]. In addition to methodological differences of the studies, educational status variation, cultural difference and their attitude difference might be attributed to this difference.

In other ways, this finding was laid in the range of scabies in the world which is conducted by Romani using systematic review (0.2 to 71.4%) [41]. Another study which was conducted in Guinea-Bissau was in line with this result [10]. The possible explanation could be a similarity of population growth rate as well as family size or fertility rate. In addition to this, climate and environmental condition are similar to the study where it was conducted and those studies [42, 43].

Another objective of this study was to identify factors associated with scabies infestation in Ethiopia. A findings of this study revealed that family size and any contact with scabies person were significantly associated. The odds of experiencing a scabies infestation was 3.1 times higher among families who had more than five members compared to those who had less than or equal to five member counterparts. This finding was supported by studies done in Iran, Fiji and Cameroon [9, 37, 41, 44]. The possible explanation could be related to sharing habits of bed and cloths in large family are high within a household and outside the household.

In this systematic review and meta-analysis, frequency of bathing has not associated with scabies. This result is inconsistent with a study conducted in Nigeria [12]. This difference might be due to environmental and methodological difference. The climate condition in Nigeria is hotter than Ethiopia, which is a favorable ground for scabies infestation.

These studies reported that the odds of undergoing to scabies infestation were higher among persons any contact with scabies than who hadn’t. This finding also supported by a study conducted in Ethiopia [45] and consistent with a study conducted in Nigeria [12]. The possible reason could be related to peoples in Nigeria and Ethiopia is more or less similar in income status and resource poor settings. In addition to this, socio-cultural practice is about a similar habit of sharing, cloth and bed in these countries.

### Limitations of the study

This meta-analysis has some limitations. The first limitation of this study was only English articles were considered to estimate the pooled prevalence and factors associated with scabies in Ethiopia. In addition, there were not enough studies of systematic reviews and meta-analysis of the problem to compare. Furthermore, in this review studies having a small sample size was included, which may have an effect on the estimated pooled prevalence reported. Therefore, this result might be affected social desirability bias. Lastly, in this meta-analysis study was found from only two regions of the country were represented, which may reflect non representation due to the limited number of articles included.

## Conclusion

In this study, the prevalence of scabies in Ethiopia was significantly high. Family size and any contact with scabies case were found significantly associated with scabies infestation. Frequency of bathing and hand washing with soap was not significantly associated. Therefore, based our findings, we recommend for Ethiopian Ministry of Health to include scabies infestation prevention and control strategies under routine health care packages and to advocate families to have a separate room to overcome overcrowding. In addition, we recommend health care professionals to give health information to the community to improve the awareness towards the mode of transmission of scabies infestation i.e. to avoid causal skin contact with scabies case.

## Data Availability

All data will be accessible form the correspondence author for a reasonable request.

## Abbreviations

CI: Confidence Interval
JBI: Joanna Briggs Institute
OR: Odds ratio
PRISMA: Preferred Reporting Items for Systematic Reviews and Meta-Analysis
SE: Standard Error
SNNPR: Southern Nations, Nationalities, and Peoples’ Region of Ethiopia
WHO: World Health Organization
